# Infodemic management for public health practitioners: landscape analysis and practical tools

**DOI:** 10.1093/eurpub/ckac129.644

**Published:** 2022-10-25

**Authors:** A Ishizumi, T Purnat, R Ludolph, S Cecchini, B Yau, C Bertrand-Ferrandis, S Briand, T Nguyen

**Affiliations:** Epidemic and Pandemic Preparedness and Prevention, WHO, Geneva, Switzerland

## Abstract

**Issue:**

Infodemics (i.e., overflow of information in physical and digital spaces that makes it difficult for people to make good health decisions) can undermine emergency response, but capacity for infodemic management has been limited in countries thus far. Specifically, there is a need to build capacities in the field with practical and scalable tools.

**Description of the problem:**

WHO has developed tools and trainings to quickly build and enhance infodemic management (IM) capacity at the country-level, such as tools for rapid generation of IM insights and a framework for conducting landscape analyses to establish sustainable IM capacities. These were developed in collaboration with multidisciplinary experts who provided feedback. We sought to create tools that can be a basis for introducing evidence-generation in health information systems to inform emergency preparedness and response, and mainstream methods into routine infodemic diagnostics activities.

**Results:**

The tools and trainings provide a comprehensive framework for diagnosing and addressing infodemics, such as a public health taxonomy to guide digital intelligence analysis and integrated analysis methods for generation of actionable insights. Additionally, the landscape analysis framework outlines steps for assessing strategic needs and assets for routinizing IM functions as part of existing public health systems and programs.

**Lessons:**

The tools and trainings will be deployed in the field to evaluate utility. Feedback from users in the global WHO infodemic manager community will be systematically captured.

**Key messages:**

• Field responders need practical tools and trainings that guide quick infodemic response during health emergencies.

• These tools and trainings can be used to diagnose and intervene on infodemics, even in settings where infodemic insights units are not yet established.

